# Biomarkers in the development of individualized treatment regimens for colorectal cancer

**DOI:** 10.3389/fmed.2022.1062423

**Published:** 2022-11-30

**Authors:** Madison Crutcher, Scott Waldman

**Affiliations:** ^1^Department of Surgery, Thomas Jefferson University, Philadelphia, PA, United States; ^2^Department of Pharmacology and Experimental Therapeutics, Thomas Jefferson University, Philadelphia, PA, United States

**Keywords:** adjuvant chemotherapy, targeted therapies, colorectal cancer, individualized medicine, biomarkers

## Abstract

**Introduction:**

Colorectal cancer (CRC) is the third most common and second most deadly malignancy in the world with an estimated 1. 9 million cases and 0.9 million deaths in 2020. The 5-year overall survival for stage I disease is 92% compared to a dismal 11% in stage IV disease. At initial presentation, up to 35% of patients have metastatic colorectal cancer (mCRC), and 20–50% of stage II and III patients eventually progress to mCRC. These statistics imply both that there is a proportion of early stage patients who are not receiving adequate treatment and that we are not adequately treating mCRC patients.

**Body:**

Targeted therapies directed at CRC biomarkers are now commonly used in select mCRC patients. In addition to acting as direct targets, these biomarkers also could help stratify which patients receive adjuvant therapies and what types. This review discusses the role of RAS, microsatellite instability, HER2, consensus molecular subtypes and ctDNA/CTC in targeted therapy and adjuvant chemotherapy.

**Discussion:**

Given the relatively high recurrence rate in early stage CRC patients as well as the continued poor survival in mCRC patients, additional work needs to be done beyond surgical management to limit recurrence and improve survival. Biomarkers offer both a potential target and a predictive method of stratifying patients to determine those who could benefit from adjuvant treatment.

## Introduction

Colorectal cancer (CRC) is the third most common and second most deadly malignancy in the world with an estimated 1.9 million cases and 0.9 million deaths in 2020 (1). With improved screening and enhanced surgical options, the overall survival in patients with CRC has improved over time with a current overall relative survival of 65% at 5 years (2). However, this survival varies greatly as the disease progresses. The 5-year overall survival for stage I disease is 92% compared to a dismal 11% in stage IV disease (3). At initial presentation up to 35% of patients have metastatic colorectal cancer (mCRC) with 20–50% of stage II and III patients eventually progressing to mCRC (4). Current recommendations suggest that patients with stage III (lymph node-positive) colon cancer undergo surgical resection followed by adjuvant chemotherapy. There continues to be controversy about the survival benefit of chemotherapy in patients with stage II disease (5). The intention of adjuvant chemotherapy is to decrease the chances of recurrence in the setting of curative resection. As stage II disease is node-negative, there is a presumption of local disease without metastases. Current recommendations suggest that stage II patients do not receive adjuvant therapy, however up to 23% will have a recurrence within 5 years indicating we are not currently capturing a population who may indeed have initial early spread and would benefit from additional therapy (6). Therefore, some argue that “high risk” stage II patients should receive adjuvant therapy in hopes of rescuing this population who will eventual relapse. Some high risk factors in stage II disease that have been suggested as warranting adjuvant treatment include T4 tumors, < 12 lymph nodes harvested at surgery, presence of bowel obstruction or perforation, poorly differentiated tumors, and the presence of lymphovascular/perineural invasion (7). Of these, only T4 disease has been validated to help identify the subset of stage II patients who benefit from adjuvant chemotherapy (8).

Standard adjuvant treatment regimens for high risk stage II or stage III disease include combination therapies of CAPEOX (capecitabine and oxaliplatin) and FOLFOX (leucovorin, fluorouracil (5-FU), and oxaliplatin). However, only 20% of patients benefit from adjuvant chemotherapy, exposing 80% of patients to unnecessary toxicity (9). In addition to these combination therapies of classic chemotherapy agents, newer targeted agents exist and may confer benefits in specific patient populations. Better biomarkers that stratify patient risk (prognostic) and predict therapeutic responses (predictive) could reduce the exposure of patient populations to unnecessary toxicity and increase the likelihood of eliminating the chance of recurrence in patients after resection. Biomarkers could aid in defining the optimum regimen of adjuvant chemotherapy, the duration of treatment, the utility of additional targeted treatments, and which patient populations should receive it (Table 1).

**Table 1:** Emerging and established biomarkers.

**Biomarker**	**Targeted drugs**	**Resistance**	**Chemotherapy**
Microsatellite instability	Pembrolizumab Nivolumab (PD-1 inhibitors) Ipilimumab (CTLA4 inhibitor)		Stage II dMMR patients have not been shown to benefit from 5-FU adjuvant therapy Oxaliplatin may have a benefit in MMR tumors
RAS	Small molecules targeting G12C variant	Confers anti-EGFR agent resistance	
BRAF	BRAF inhibitors	Negative predictor of response to anti-EGFR therapies	
HER2	Trastuzumab Lapatinib Pertuzumab Trastuzumab deruxtecan	Predict resistance to anti-EGFR therapies	HER2 high patients may benefit from adjuvant chemotherapy
APC	Tankyrase inhibitors		
CEA			CEA high patients may benefit from adjuvant chemotherapy
NTRK	Enterctinib Larotrectinib		

Biomarkers offer targets for directed drug therapy as well as potential markers of resistance. In addition, biomarkers can be used to help guide chemotherapy decisions.

## Microsatellite instability

High microsatellite instability (MSI-H) is the phenotype of a deficient mismatch repair (dMMR) system and is present in about 15% of colorectal cancers. Microsatellites are short tandem repeats of single nucleotide or di-, tri-, or tetra-nucleotides in DNA sequences found throughout the tumor genome and are a marker of a hypermutable phenotype. The mismatch repair (MMR) system functions to rectify errors that may occur during DNA replication. With the inactivation of at least one of the DNA MMR genes (MLH1, MSH2, MSH6, and PMS2) through either mutations or transcriptional silencing, the MMR system is unable to function leading to an accumulation of errors in the DNA (10). This inactivation stems from either germline mutations in the MMR genes themselves or somatic hypermethylation of CpG islands surrounding the promotor region in the genes. Germline mutations in MMR lead to hereditary non-polyposis colorectal cancer (HNPCC or Lynch Syndrome) which causes ~3% of all CRCs (11). The somatic hypermethylation of CpG islands is known as the CpG island methylator phenotype (CIMP). These CIMP tumors comprise the majority of sporadic MSI-H CRCs (12). These CIMP tumors are in contrast to tumors with chromosomal instability (CIN) which follow the more traditional pathway of initial APC mutation causing a tubular adenoma with subsequent accumulated mutations leading to cancer (13).

MSI-H/dMMR is more common among stage II tumors compared with stage III CRCs and relatively uncommon in stage IV (metastatic) CRCs suggesting MSI-H/dMMR tumors are less likely to metastasize. Indeed, MSI-H/dMMR is independently associated with improved survival compared with tumors with proficient MMR (pMMR) (14). In addition, MSI-H/dMMR tumors also have lower recurrence rates compared with pMMR tumors (15). It has also been shown that MSI-H/dMMR tumors do not respond well to 5-FU-based adjuvant chemotherapy (16). Indeed, cells require a competent MMR system for 5-FU to be effective (17). Current recommendations suggest that patients with stage II colon cancer with MSI-H/dMMR should not receive adjuvant 5-FU-based chemotherapy based on this known favorable prognosis and lack of response to therapy.

Unlike 5-FU, oxaliplatin leads to DNA-cross linking and inhibits DNA synthesis and transcription. This damage is not recognized by the MMR system and dMMR tumors should not be resistant to oxaliplatin. The MOSAIC trial revealed improvement in 5-year DFS and 6-year OS for stage III colon cancers with the addition of oxaliplatin to 5-FU regardless of MMR status (18). Ten year follow up of the MOSAIC trial confirmed the benefit of oxaliplatin as adjuvant therapy in stage II/III colon cancers. More recent work has revealed a potential benefit to the addition of oxaliplatin to fluoropyrimidines in adjuvant chemotherapy for MSI-H stage III colon cancer (19).

In addition to standard chemotherapy, additional treatment options exist that may specifically benefit in MSI-H/dMMR patients. As previously discussed, MSI-H/dMMR have a baseline improved clinical course compared to tumors with pMMR. This may be due to their hypermutable phenotype contributing to the production of abnormal peptides that serve as neoantigens, producing specific antitumor immune responses leading to decreased tumor growth and metastasis (20). Sporadic MSI-H CRC have a distinct phenotype that includes right colon predominance, increased prevalence in women and poor differentiation/mucinous histology. MSI-H tumors also exhibit an elevated number of tumor-infiltrating lymphocytes (TILs), supporting this neoantigen hypothesis (21). This baseline local immune control contributes to improved patient survival in MSI-H CRC and also sensitizes tumors in these patients to immune checkpoint inhibitors targeting either programmed cell death-1 protein (PD-1) or cytotoxic T-lymphocyte-associated protein 4 (CTLA-4). PD-1 is expressed on T cells, and binding of its ligands (PD-L1 and PD-L2) downregulates T cell effector function. In that context, tumors can escape immune detection by upregulating expression of programmed death ligand 1 (PD-L1) (22). Inhibitors of PD-1 block the receptor from interacting with its ligands, promoting tumor cell killing by effector T cells. Inhibitors of PD-1, pembrolizumab (Keytruda) and nivolumab (Opdivo), are FDA-approved for patients with mCRC with dMMR or MSI-H and confer a significant survival benefit when used (23, 24). An additional target, CTL-4, is transiently expressed on activated T cells with its expression inhibiting the production of cytokines and providing a negative feedback signal to T cells prompting T cell cycle arrest. Inhibition of CTLA-4 may lead to reactivation of T cells allowing them to overcome tumor-induced immune tolerance (25). Ipilimumab (Yervoy) is an anti-CTLA-4 antibody used in metastatic dMMR/MSI-H patients in combination with nivolumab (26). This combination of nivolumab and low-dose ipilimumab produced an objective response rate of 64%, complete response rate of 9%, and disease control rate of 84%, all of which were durable (27). While the results of immune checkpoint blockade in dMMR/MSI-H CRC patients have been encouraging, single agent checkpoint inhibitors are not efficacious in patients with pMMR which makes up the majority of CRC patients (28). In addition, while immune checkpoint inhibitors are approved in mCRC dMMR/MSI-H disease, their utility as adjuvant therapy in localized disease and their efficacy in combination are being explored (29, 30). The use of dMMR/MSI-H as a biomarker in determining the need for adjuvant therapy, the type of adjuvant chemotherapy and the addition of an immune checkpoint inhibitor could better optimize the alignment of treatment groups and therapies.

## MAPK pathway (Ras-Raf-MEK-ERK)

Gain or loss of function mutations in proteins in the mitogen-activated protein kinase (MAPK) pathway lead to dysregulated proliferative cell signaling ultimately driving tumorigenesis. The first protein to be activated in the pathway is RAS, a commonly mutated protein in CRC (31). In the normal cell, activation of RAS begins with an extracellular ligand binding to a receptor-linked tyrosine kinase like epidermal growth factor receptor (EGFR). This binding activates the tyrosine kinase in the cytoplasmic domain of the receptor causing phosphorylation of EGFR and interaction with RAS. This triggers RAS, a GTPase, to exchange a GDP molecule for GTP, activating the pathway and initiating a kinase cascade leading to the activation of Raf, MAPK/ERK (MEK1 or 2) and ultimately MAPK (32).

RAS (KRAS, NRAS, and HRAS) is the most frequently mutated gene family in cancers with the most common oncogenic mutant of the RAS family being KRAS G12C. KRAS mutations are present in 30–50% of CRC with NRAS mutated in 3–5% and HRAS mutated in < 1% (33, 34). KRAS mutations account for up 45% of mCRC and ~15–37% of early stage tumors (35, 36). Historically, RAS was considered “undruggable” due to its picomolar affinity for GTP/GDP, the absence of identified allosteric regulatory sites, and the necessity of wild type RAS in normal biologic functions. However, small molecules that specifically inhibit the G12C variant have been identified, making RAS a potential therapeutic target (37).

Monoclonal antibodies targeting EGFR, including cetuximab and panitumumab, are routinely used in mCRC. These monoclonal antibodies compete with the endogenous EGFR ligand and after binding, block phosphorylation, leading to internalization and degradation of the receptor. Cetuximab has been approved as a first-line treatment in mCRC patients with wild-type KRAS in combination with chemotherapy (38). Unfortunately, the addition of cetuximab to FOLFOX failed to improve disease-free or overall survival in post-resection stage III colon cancer patients (39). There is emerging evidence of the effectiveness of combining EGFR and KRAS G12C inhibitors in advanced disease. EGFR signaling has been identified as the primary mechanism of resistance to KRAS G12C inhibitors and this combination may overcome this resistance (40). The combination of anti-EGFR and KRAS G12C inhibitors is effective in cell lines, patient-derived organoids, and xenografts (41).

One downstream effector target of RAS is the RAF family, made up of c-RAF1, BRAF, and ARAF. Of these, BRAF is the most frequently mutated in tumors (42). Outside the constitutive activation of RAS, mutations in codon 600 of the BRAF gene produce RAS-independent activation of the MAPK pathway, leading to increased cell proliferation and survival. Sporadic MSI CRCs often show increased co-occurrence of BRAFV600E mutations compared to CRCs overall (43). These somatic BRAFV600E mutations increase BRAF/MEK/ERK signaling leading to the CIMP which silences MLH1, ultimately resulting in dMMR. The presence of a BRAF mutation indicates a sporadic MSI tumor and virtually excludes the diagnosis of Lynch syndrome (44). Patients with BRAF mutations experience poorer survival compared to patients with wild-type BRAF (45). BRAF mutations are associated with more right-sided primary tumors and with an increased risk of metastasis to the peritoneum and distant lymph nodes (46). BRAF and KRAS mutations are not coincident in tumors, and many KRAS wild type mCRC have BRAF mutations. These mutations identify tumors that are unresponsive to anti-EGFR therapies when combined with chemotherapy (47).

BRAF inhibitors are used extensively in BRAFV600E melanomas with positive treatment results (48). While BRAF inhibitor monotherapy in BRAFV600 melanoma leads to response rates of >50%, only ~5% of BRAFV600 CRC patients respond (49). Since EGFR mediates resistance to BRAF inhibitors, the differing expression of EGFR in CRC, compared to melanoma, may explain this difference in response rates. In CRC, BRAF inhibition leads to feedback activation of EGFR which increases proliferation even in the presence of BRAFV600 inhibition (50). In contrast, simultaneous blockade of EGFR and BRAF produced synergistic inhibition of tumor growth in murine CRC models through enhanced MAPK suppression (51). Dual treatment with EGFR and BRAF inhibitors in previously-treated patients with BRAF V600E mCRC improved overall survival and progression-free survival compared to standard chemotherapy (52). Moreover, triple therapy inhibiting BRAF, EGFR, and MEK is effective against BRAFV600 tumors (53, 54).

## HER2

HER2 (human epidermal growth factor receptor 2) is a transmembrane receptor that acts as an intracellular tyrosine kinase. Homo- or heterodimerization of HER2 with an additional member of the EGFR family (EGFR/HER2/ERBB) leads to the activation of either the RAS-RAF-ERK or PI3K-PTEN-AKT pathway leading to increased cellular proliferation. The amplification of the HER2 gene or overexpression of the HER2 protein has been targeted in solid tumor malignancies other than CRC. While therapies that block HER2 (trastuzumab, lapatinib, and pertuzumab) have gained prominence in treating patients with HER2-overexpressing tumors in these other malignancies, there are no HER-2-directed therapies approved by the FDA to treat CRC.

**Figure 1:**
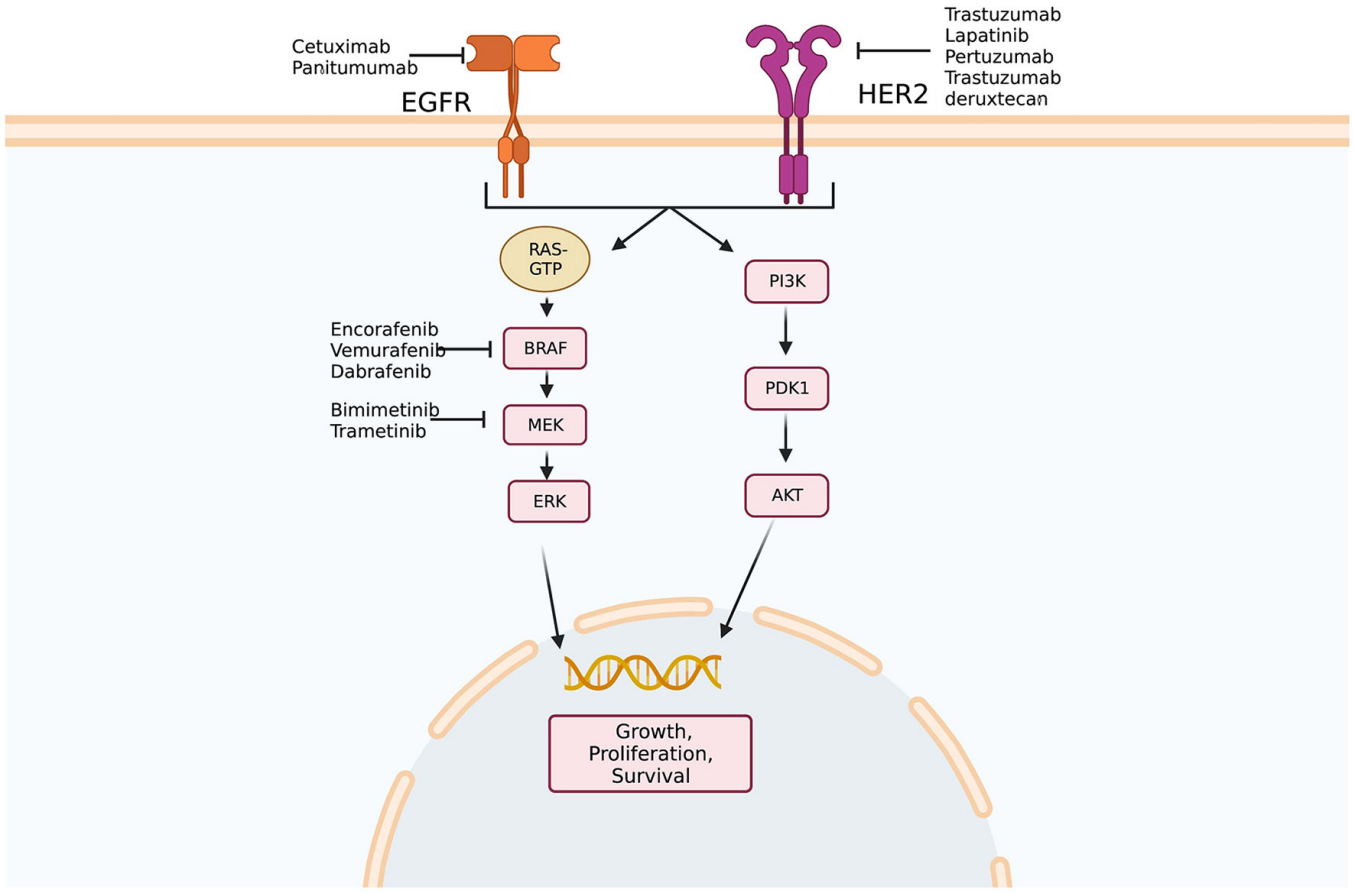
Epidermal growth factor signaling pathways. Multiple potential targets for therapy exist along epidermal growth factor receptor (EFGR) and human epidermal growth factor receptor 2 (HER2) pathways. In addition, amplification of HER2 has been implicated in anti-EGFR therapy resistance as activation of the HER2 pathway may bypass blockade of EGFR. Created with BioRender.com. Adapted from Crutcher et al. (37) Overview of predictive and prognostic biomarkers and their importance in developing a clinical pharmacology treatment plan in colorectal cancer patients, Expert Review of Clinical Pharmacology.

Preclinical work initially showed that HER2-amplified tumors were responsive to dual HER2-directed therapies, but not individual agents alone. Using this information, a phase 2 trial examining dual HER2 therapy comprising a tyrosine kinase inhibitor and anti-HER2 monoclonal antibody in KRAS wild-type, HER2-positive mCRC patients demonstrated that 30% of patients had objective responses and 44% had stable disease (55). A phase 2 trial of trastuzumab deruxtecan, a HER2-targeted antibody-drug conjugate, in patients who had previously progressed on at least two previous treatment regimens, showed an objective response rate of 45.3% (56). In quadruple WT populations (KRAS, NRAS, BRAF, and PIK3CA WT) treated with anti-EGFR therapies, the HER2 pathway may function as a bypass leading to resistance to anti-EGFR agents (57) (Figure 1). Indeed, HER2 expression predicts unresponsiveness to EGFR-targeted therapies in mCRC (58).

In addition to predicting response to HER2 and EGFR directed therapies, HER2 expression could help identify which patients may have a benefit from adjuvant chemotherapy. One study showed that among HER2 high patients, those who received chemotherapy had better OS and DFS than chemotherapy naïve patients. They showed no difference in outcomes among chemo-treated and chemo-naïve patients in the HER2 low group (59). This implies HER2 expression in CRC can be used as a direct target as well as a biomarker of resistance, and even eventually a guide in chemotherapy.

## APC

In most CRCs Wnt/β-catenin signaling is activated by loss-of-function mutations in the adenomatous polyposis coli (APC) gene. The β-catenin-dependent Wnt signaling pathway is initiated by the binding of secreted cysteine-rich Wnt glycoproteins to LRP5/6 receptors and FZD receptors. The secretion of Wnt ligands depends on acylation by Porcupine (PORCN) (60). Binding of the Wnt ligands to LRP5/6 and FZD receptors on the cell surface induces disheveled (DVL) which leads to suppression of glycogen synthase kinase 3β (GSK3β). Together GSK3β, axin, and casein kinase 1 (CK1a) form a destruction complex which is stabilized by APC and phosphorylates β-catenin, priming it for degradation by the ubiquitin-proteosome pathway. In the presence of Wnt, and suppression of GSK3β, un-phosphorylated β-catenin accumulates in the cytosol, translocates to the nucleus, and interacts with TCF/LEF transcription factors to trigger expression of Wnt targets like c-Myc, cyclin D1, and CDKN1A (61). Inactivating mutations of APC de-stabilize the destruction complex, leading to activation of the Wnt signaling pathway which drives tumorigenesis.

While dysregulation of the Wnt/β-catenin signaling pathway is common in CRCs, this pathway lacks druggable molecular targets. Tankyrases (TNKSs) are members of poly-ADP-ribose polymerases (PARPs) family that poly-ADP-ribosylate and downregulate axins resulting in an overexpression of β-catenin. Tankyrase inhibitors (TNKSi) have been developed as potential therapeutic agents in CRC (62). APC may effect response to tankyrase inhibitors. It has been shown that drug-sensitive CRC cells had truncated forms of APC that lacked all seven β-catenin-binding 20-amino acid repeats (AARs) resulting in cell response to TNKSi. Conversely, drug-resistant CRC cells had longer forms of APCs with two of more 20AARs (63). Identification of APC status could be prognostic in determining potential response to TNKSi.

## CEA

Carcinoembryonic antigen (CEA) is a cell adhesion molecule that is elevated in the serum of patients with a variety of cancers, including CRC. CEA levels have been used postoperatively in surveillance and higher preoperative CEA levels have been shown to be an independent predictor of both overall and disease-free survival rates. In addition, patients with node-negative colon cancer but elevated preoperative CEA levels have a poor prognosis similar to those with node-positive disease (64). These patients may be candidates for adjuvant chemotherapy. As previously discussed stage II colon cancers do not typically receive adjuvant chemotherapy. However those stage II patients with high risk features may benefit from adjuvant therapy but there has been difficulty in defining this group. Studies have shown that CEA levels could potentially be used to risk stratify stage II patients and inform treatment decisions (65).

## NTRK

Neurotrophic tyrosine receptor kinase (NTRK) gene fusions are extremely rare in CRC occurring in less than 1% of tumors (66). However, they are more frequently found in patients with dMMR (67). The FDA has approved two tropomyisin receptor kinase (TRK) inhibitors, entrectinib, and larotrectinib, for use in patients with NTRK fusion-bearing cancers in either a worsening metastatic setting or locally advanced unresectable tumors (68). This is an example of tissue agnostic treatments that can be used in any solid tumor, not just CRC.

## Consensus molecular subtypes

An additional method of categorizing CRCs that may help guide treatment decisions are the Consensus Molecule Subtypes (CMS). CMS1 or MSI immune tumors account for 14% of CRCs. They have a high rate of mutations, with frequent BRAF mutations, and sizeable immune infiltration. The majority of MSI tumors fall in this category and, as previously discussed, these tumors are responsive to immune checkpoint inhibitors. In addition, the BRAFV600E mutation predisposes resistance to treatment with anti-EGFR agents. CMS2 or canonical tumors make up 37% of tumors and have upregulated Wnt/Myc signaling. These tumors stem from the canonical adenoma-to-carcinoma sequence typified by the initial loss of APC, a following activating mutation in KRAS, and an ultimate loss of TP53. CMS3 or metabolic tumors comprise 13% of cases and have frequent KRAS mutations and dysregulation of cancer metabolic pathways. As discussed previously, KRAS mutation may indicate a poor response to anti-EGFR therapy. CMS4 or mesenchymal tumors form 23% of cases and are characterized by transforming growth factor beta (TGFβ) pathway activation, enhanced angiogenesis, stromal activation and inflammatory infiltrates (69).

These four molecular subtypes can be broadly divided into “hot” and “cold” CRCs based on immune infiltration. The high immune infiltration of CMS1/MSI-H tumors has been discussed, as well as their responsiveness to treatment with immune checkpoint inhibitors. While CMS4 tumors also have increased immune cell infiltrates, responses to immunotherapy may be altered by TGFβ signaling. In comparison to the anti-tumor immune environment of CMS1 tumors, the CMS4 tumor microenvironment is pro-inflammatory (70). Indeed, TGFβ may be immunosuppressive and drive immune evasion in CRC (71). Alternatively, CMS2 and CMS3 tumors are “cold” tumors reflecting low immune cell infiltrates. CMS2 and CMS3 tumors may respond to alternative immunogenic stimuli, like vaccines or co-stimulatory compounds, but do not respond to immune checkpoint inhibitors. CMS2 and CMS3 tumors also respond to anti-VEGF agents (72). CMS classification has the potential to provide prognostic information, since CMS2 and CMS3 tumors have a better prognosis than CMS1 and CMS4. One study examining CMS status among stage II CRC found adjuvant chemotherapy had no benefit in CMS1 subtype tumors, and a significant decrease in DFS for CMS4 tumors (73). In contrast, stage II and III patients with either the CMS2 or CMS3 have benefit from adjuvant therapy (74). While not currently used in clinical practice CMS subtypes may eventually help guide targeted and chemotherapy decisions.

## Circulating tumor cells (CTCs) and circulating tumor DNA (ctDNA)

The concept of a “liquid biopsy” for solid tumors has recently emerged, reflecting sampling convenience and its ability to capture the varying molecular markers of a solid tumor. Liquid biopsies have multiple advantages over tissue biopsies, such as assessing molecularly divergent metastatic lesions, capturing the heterogeneity of a tumor, and evaluating potential resistance mutations in real time as treatment progresses. Circulating tumor cells (CTCs) are individual, or clusters of, cancer cells circulating in the bloodstream resulting from passive shedding or intravasation from the primary lesion or metastases (75). The amount of detectable CTCs detected is associated with treatment outcomes and overall survival (76). In contrast to CTCs, cell-free circulating tumor DNA (ctDNA) comprises somatic and epigenetic DNA alterations from tumor cells released into bloodstream following apoptosis or necrosis. ctDNA is more abundant within the bloodstream than CTCs but both can be detected and interrogated for actionable treatment targets and emergent resistant sub-clones, therefore assisting in treatment decisions before and after initiation of therapy.

There is an established relationship between ctDNA and tumor burden, with ctDNA positivity increasing with CRC stage (77). In this sense, ctDNA could identify high risk early stage patients. In addition, as discussed earlier, there are several biomarkers that can predict prognosis or treatment response in CRC such as MSI-H/dMMR (susceptibility to immune checkpoint inhibitors) as well as KRAS/BRAF (anti-EGFR resistance). A study interrogating the emergence of mutated KRAS alleles in ctDNA during anti-EGFR therapy revealed that these alleles decline when treatment is suspended, demonstrating that liquid biopsies can be used to track treatment resistance (78). The ability to accurately capture these markers prior to the initiation treatment could help tailor therapeutic planning. Furthermore, the ability to track these markers during treatment could both ensure treatment response and monitor for developing resistance.

Currently, there is controversy as to what proportion of stage II CRC patients should receive adjuvant therapy. While some high risk characteristics have been suggested, these are not validated and there is no consensus (79). In stage II CRC, post-operative patients who were positive for ctDNA were at extremely high risk for recurrence when not treated with adjuvant chemotherapy (80, 81). A study surveying ctDNA status in patients after curative-intent surgery revealed that 100% of patients with ctDNA detected after treatment completion ultimately recurred (82). In patients with resectable colorectal liver metastases, patients with ctDNA detected after surgery had a significantly poorer relapse-free survival and overall survival. In addition, all patients with persistently detectable ctDNA after adjuvant chemotherapy recurred (83). A study in stage I-III patients revealed that in the majority, ctDNA identified relapse after definitive treatment. The same study also showed that ctDNA status was independently associated with relapse after adjusting for other clinicopathologic risk factors (84). ctDNA could potentially be used as an adjunct to the traditional TNM staging and other potential prognostic markers in determining which patients receive adjuvant therapy.

## Summary

Despite improvements in screening and surgical interventions, CRC has remained the second most common cause of cancer-related death in the United States. While it has an overall favorable relative survival 5 year survival of 65%, inadequacies in treatments are revealed when stage by stage prognosis is examined (2). The 5-year overall survival for stage I disease (small, no lymph node spread) is 92% compared to 11% in stage IV (metastatic) (3). Approximately 35% of patients have metastatic disease at initial presentation with 20–50% of stage II and stage III patients eventually progressing to metastatic disease (4). These survival statistics illuminate multiple areas for improvement in the treatment of CRC. The high recurrence rates among patients who present with localized disease indicates missed opportunities for curative treatment in some patient populations. Currently, adjuvant therapy is consistently given to patients with stage III disease (positive lymph nodes) with some controversy in stage II disease. Again, the high recurrence rates among this population suggest there could be additional benefit from adjuvant treatment.

Further, much like innovations in targeted therapy, strides have been made in novel sampling techniques. ctDNA in the blood of CRC patients reflects the entire tumor genome. Increasing levels of ctDNA have been shown to be correlated with worse survival showing ctDNA could potentially be included in staging algorithms (85). In addition to sampling at diagnosis in order to stage and determine molecular markers, ctDNA levels and mutation expression can be followed to monitor for recurrence or emerging treatment resistance. While CMS subtypes currently are not recommended for use in clinical practice, this may change as a greater understanding of their biology emerges.

## Author contributions

Review of relevant papers and manuscript preparation: MC and SW. All authors reviewed the results and approved the final version of the manuscript.

## Funding

This work was supported by the National Institutes of Health (R01 CA204881, R01 CA206026, and P30 CA56036), the Defense Congressionally Directed Medical Research Program W81XWH-17-PRCRP-TTSA, a grant from the Courtney Anne Diacont Memorial Foundation, Lorraine and David Swoyer, and Targeted Diagnostic & Therapeutics, Inc. to SW. MC was supported by NIH institutional award T32 GM008562 for Post-doctoral Training in Clinical Pharmacology. SW is the Samuel M.V. Hamilton Professor of Thomas Jefferson University.

## Conflict of interest

SW is the Chair of the Scientific Advisory Board and member of the Board of Directors of Targeted Diagnostics & Therapeutics, Inc. which provided research funding that, in part, supported this work and has a license to commercialize inventions related to this work. The remaining author declares that the research was conducted in the absence of any commercial or financial relationships that could be construed as a potential conflict of interest.

## Publisher's note

All claims expressed in this article are solely those of the authors and do not necessarily represent those of their affiliated organizations, or those of the publisher, the editors and the reviewers. Any product that may be evaluated in this article, or claim that may be made by its manufacturer, is not guaranteed or endorsed by the publisher.

## References

[1] Xi Y Xu P. Global colorectal cancer burden in 2020 and projections to 2040. Transl Oncol. (2021) 14:101174. 10.1016/j.tranon.2021.101174 34243011 PMC8273208

[2] Siegel RL Miller KD Fuchs HE Jemal A. Cancer statistics, 2022. CA Cancer J Clin. (2022) 72:7–33. 10.3322/caac.21708 35020204

[3] Surveillance Epidemiology and End Results Program. Available online at: https://seer.cancer.gov/ (accessed March 14, 2022).

[4] Zacharakis M Xynos ID Lazaris A Smaro T Kosmas C Dokou A. Predictors of survival in stage IV metastatic colorectal cancer. Anticancer Res. (2010) 30:653–60. 20332485

[5] Babcock BD Aljehani MA Jabo B Choi AH Morgan JW Selleck MJ. High-Risk Stage II Colon Cancer: Not All Risks Are Created Equal. Ann Surg Oncol. (2018) 25:1980–5. 10.1245/s10434-018-6484-8 29675762

[6] Wilkinson NW Yothers G Lopa S Costantino JP Petrelli NJ Wolmark N. Long-term survival results of surgery alone versus surgery plus 5-fluorouracil and leucovorin for stage II and stage III colon cancer: pooled analysis of NSABP C-01 through C-05. A baseline from which to compare modern adjuvant trials. Ann Surg Oncol. (2010) 17:959–66. 10.1245/s10434-009-0881-y 20082144 PMC2935319

[7] Chan GHJ Chee CE. Making sense of adjuvant chemotherapy in colorectal cancer. J Gastrointest Oncol. (2019) 10:1183–92. 10.21037/jgo.2019.06.03 31949938 PMC6954995

[8] Kumar A Kennecke HF Renouf DJ Lim HJ Gill S Woods R. Adjuvant chemotherapy use and outcomes of patients with high-risk versus low-risk stage II colon cancer. Cancer. (2015) 121:527–34. 10.1002/cncr.29072 25332117

[9] Auclin E Zaanan A Vernerey D Douard R Gallois C Laurent-Puig P. Subgroups and prognostication in stage III colon cancer: future perspectives for adjuvant therapy. Ann Oncol. (2017) 28:958–68. 10.1093/annonc/mdx030 28453690

[10] Nojadeh JN Behrouz Sharif S Sakhinia E. Microsatellite instability in colorectal cancer. Excli J. (2018) 17:159–68. 29743854 10.17179/excli2017-948 PMC5938532

[11] Cerretelli G Ager A Arends MJ Frayling IM. Molecular pathology of Lynch syndrome. J Pathol. (2020) 250:518–31. 10.1002/path.5422 32141610

[12] Advani SM Swartz MD Loree J Davis JS Sarsashek AM Lam M. Epidemiology and Molecular-Pathologic Characteristics of CpG Island Methylator Phenotype (CIMP) in Colorectal Cancer. Clin Colorectal Cancer. (2021) 20:137–47.e1. 10.1016/j.clcc.2020.09.007 33229221

[13] Fearon ER Vogelstein B. A genetic model for colorectal tumorigenesis. Cell. (1990) 61:759–67. 10.1016/0092-8674(90)90186-I 2188735

[14] Sargent DJ Shi Q Yothers G Tejpar S Bertagnolli MM Thibodeau SN. Prognostic impact of deficient mismatch repair (dMMR) in 7,803 stage II/III colon cancer (CC) patients (pts): A pooled individual pt data analysis of 17 adjuvant trials in the ACCENT database. JCO. (2014) 32:3507–3507. 10.1200/jco.2014.32.15_suppl.3507

[15] Battaglin F Naseem M Lenz H-J Salem ME. Microsatellite instability in colorectal cancer: overview of its clinical significance and novel perspectives. Clin Adv Hematol Oncol. (2018) 16:735–45. 30543589 PMC7493692

[16] Sun BL. Current microsatellite instability testing in management of colorectal cancer. Clin Colorectal Cancer. (2021) 20:e12–20. 10.1016/j.clcc.2020.08.001 32888812

[17] Jover R Zapater P Castells A Llor X Andreu M Cubiella J. The efficacy of adjuvant chemotherapy with 5-fluorouracil in colorectal cancer depends on the mismatch repair status. Eur J Cancer. (2009) 45:365–73. 10.1016/j.ejca.2008.07.016 18722765

[18] André T Boni C Navarro M Tabernero J Hickish T Topham C. Improved overall survival with oxaliplatin, fluorouracil, and leucovorin as adjuvant treatment in stage II or III colon cancer in the MOSAIC trial. J Clin Oncol. (2009) 27:3109–16. 10.1200/JCO.2008.20.6771 19451431

[19] Cohen R Taieb J Fiskum J Yothers G Goldberg R Yoshino T. Microsatellite instability in patients with stage III colon cancer receiving fluoropyrimidine with or without oxaliplatin: an ACCENT pooled analysis of 12 adjuvant trials. J Clin Oncol. (2021) 39:642–51. 10.1200/JCO.20.01600 33356421 PMC8189604

[20] Guidoboni M Gafà R Viel A Doglioni C Russo A Santini A. Microsatellite instability and high content of activated cytotoxic lymphocytes identify colon cancer patients with a favorable prognosis. Am J Pathol. (2001) 159:297–304. 10.1016/S0002-9440(10)61695-1 11438476 PMC1850401

[21] De' Angelis GL Bottarelli L Azzoni C De' Angelis N Leandro G Di Mario F. Microsatellite instability in colorectal cancer. Acta Biomed. (2018) 89:97–101. 30561401 10.23750/abm.v89i9-S.7960 PMC6502181

[22] Piawah S Venook AP. Targeted therapy for colorectal cancer metastases: A review of current methods of molecularly targeted therapy and the use of tumor biomarkers in the treatment of metastatic colorectal cancer. Cancer. (2019) 125:4139–47. 10.1002/cncr.32163 31433498

[23] Le DT Uram JN Wang H Bartlett BR Kemberling H Eyring AD. PD-1 Blockade in Tumors with Mismatch-Repair Deficiency. N Engl J Med. (2015) 372:2509–20. 10.1200/jco.2015.33.15_suppl.lba100 26028255 PMC4481136

[24] Overman MJ McDermott R Leach JL Lonardi S Lenz H-J Morse MA. Nivolumab in patients with metastatic DNA mismatch repair-deficient or microsatellite instability-high colorectal cancer (CheckMate 142): an open-label, multicentre, phase 2 study. Lancet Oncol. (2017) 18:1182–91. 10.1016/S1470-2045(17)30422-9 28734759 PMC6207072

[25] Fiegle E Doleschel D Koletnik S Rix A Weiskirchen R Borkham-Kamphorst E. Dual CTLA-4 and PD-L1 blockade inhibits tumor growth and liver metastasis in a highly aggressive orthotopic mouse model of colon cancer. Neoplasia. (2019) 21:932–44. 10.1016/j.neo.2019.07.006 31412307 PMC6700499

[26] Igaue S Okuno T Ishibashi H Nemoto M Hiyoshi M Kawasaki H. A pathological complete response after nivolumab plus ipilimumab therapy for DNA mismatch repair-deficient/microsatellite instability-high metastatic colon cancer: A case report. Oncol Lett. (2022) 24:211. 10.3892/ol.2022.13332 35720492 PMC9178690

[27] Lenz H-J Van Cutsem E Luisa Limon M Wong KYM Hendlisz A Aglietta M. First-Line Nivolumab Plus Low-Dose ipilimumab for microsatellite instability-high/mismatch repair-deficient metastatic colorectal cancer: the phase II CheckMate 142 study. J Clin Oncol. (2021) 40:161–70. 10.1200/JCO.21.01015 34637336

[28] Overman MJ Ernstoff MS Morse MA. Where we stand with immunotherapy in colorectal cancer: deficient mismatch repair, proficient mismatch repair, and toxicity management. Am Soc Clin Oncol Educ Book. (2018) 38:239–47. 10.1200/EDBK_200821 30231358

[29] Chalabi M Fanchi LF Dijkstra KK Van den Berg JG Aalbers AG Sikorska K. Neoadjuvant immunotherapy leads to pathological responses in MMR-proficient and MMR-deficient early-stage colon cancers. Nat Med. (2020) 26:566–76. 10.1038/s41591-020-0805-8 32251400

[30] Overman MJ Lonardi S Wong KYM Lenz H-J Gelsomino F Aglietta M. Durable Clinical Benefit With Nivolumab Plus Ipilimumab in DNA Mismatch Repair-Deficient/Microsatellite Instability-High Metastatic Colorectal Cancer. J Clin Oncol. (2018) 36:773–9. 10.1200/JCO.2017.76.9901 29355075

[31] Wan M-L Wang Y Zeng Z Deng B Zhu B-S Cao T. Colorectal cancer (CRC) as a multifactorial disease and its causal correlations with multiple signaling pathways. Biosci Rep. (2020) 40:BSR20200265. 10.1042/BSR20200265 32149326 PMC7087324

[32] Khan K Valeri N Dearman C Rao S Watkins D Starling N. Targeting EGFR pathway in metastatic colorectal cancer- tumour heterogeniety and convergent evolution. Crit Rev Oncol Hematol. (2019) 143:153–63. 10.1016/j.critrevonc.2019.09.001 31678702

[33] Serebriiskii IG Connelly C Frampton G Newberg J Cooke M Miller V. Comprehensive characterization of RAS mutations in colon and rectal cancers in old and young patients. Nat Commun. (2019) 10:3722. 10.1038/s41467-019-11530-0 31427573 PMC6700103

[34] Prior IA Hood FE Hartley JL. The frequency of ras mutations in cancer. Cancer Res. (2020) 80:2969–74. 10.1158/0008-5472.CAN-19-3682 32209560 PMC7367715

[35] Modest DP Ricard I Heinemann V Hegewisch-Becker S Schmiegel W Porschen R. Outcome according to KRAS-, NRAS- and BRAF-mutation as well as KRAS mutation variants: pooled analysis of five randomized trials in metastatic colorectal cancer by the AIO colorectal cancer study group. Ann Oncol. (2016) 27:1746–53. 10.1093/annonc/mdw261 27358379 PMC4999563

[36] Taieb J Le Malicot K Shi Q Penault-Llorca F Bouché O Tabernero J. Prognostic value of braf and kras mutations in MSI and MSS stage III colon cancer. J Natl Cancer Inst. (2017) 109:djw272. 10.1093/jnci/djw272 28040692 PMC6075212

[37] Crutcher MM Baybutt TR Kopenhaver JS Snook AE Waldman SA. Emerging drug targets for colon cancer: A preclinical assessment. Expert Opin Ther Targets. (2022) 26:207–16. 10.1080/14728222.2022.2039119 35129035 PMC9075542

[38] Jonker DJ O'Callaghan CJ Karapetis CS Zalcberg JR Tu D Au H-J. Cetuximab for the treatment of colorectal cancer. N Engl J Med. (2007) 357:2040–8. 10.1056/NEJMoa071834 18003960

[39] Taieb J Tabernero J Mini E Subtil F Folprecht G Van Laethem J-L. Oxaliplatin, fluorouracil, and leucovorin with or without cetuximab in patients with resected stage III colon cancer (PETACC-8): an open-label, randomised phase 3 trial. Lancet Oncol. (2014) 15:862–73. 10.1016/S1470-2045(14)70227-X 24928083

[40] Patelli G Tosi F Amatu A Mauri G Curaba A Patanè DA. Strategies to tackle RAS-mutated metastatic colorectal cancer. ESMO Open. (2021) 6:100156. 10.1016/j.esmoop.2021.100156 34044286 PMC8167159

[41] Amodio V Yaeger R Arcella P Cancelliere C Lamba S Lorenzato A. EGFR blockade reverts resistance to KRASG12C inhibition in colorectal cancer. Cancer Discov. (2020) 10:1129–39. 10.1158/2159-8290.CD-20-0187 32430388 PMC7416460

[42] Lavoie H Therrien M. Regulation of RAF protein kinases in ERK signalling. Nat Rev Mol Cell Biol. (2015) 16:281–98. 10.1038/nrm3979 25907612

[43] Kambara T Simms LA Whitehall VLJ Spring KJ Wynter CVA Walsh MD. BRAF mutation is associated with DNA methylation in serrated polyps and cancers of the colorectum. Gut. (2004) 53:1137–44. 10.1136/gut.2003.037671 15247181 PMC1774130

[44] Domingo E Niessen RC Oliveira C Alhopuro P Moutinho C Espín E. BRAF-V600E is not involved in the colorectal tumorigenesis of HNPCC in patients with functional MLH1 and MSH2 genes. Oncogene. (2005) 24:3995–8. 10.1038/sj.onc.1208569 15782118

[45] Tan E Whiting J Xie H Imanirad I Carballido E Felder S. BRAF Mutations Are Associated with Poor Survival Outcomes in Advanced-stage Mismatch Repair-deficient/Microsatellite High Colorectal Cancer. Oncologist. (2022) 27:191–7. 10.1093/oncolo/oyab055 35274712 PMC8914499

[46] Van Cutsem E Cervantes A Adam R Sobrero A Van Krieken JH Aderka D. ESMO consensus guidelines for the management of patients with metastatic colorectal cancer. Ann Oncol. (2016) 27:1386–422. 10.1093/annonc/mdw235 27380959

[47] Van Cutsem E Köhne C-H Láng I Folprecht G Nowacki MP Cascinu S. Cetuximab plus irinotecan, fluorouracil, and leucovorin as first-line treatment for metastatic colorectal cancer: updated analysis of overall survival according to tumor KRAS and BRAF mutation status. J Clin Oncol. (2011) 29:2011–9. 10.1200/JCO.2010.33.5091 21502544

[48] Flaherty KT Puzanov I Kim KB Ribas A McArthur GA Sosman JA. Inhibition of mutated, activated BRAF in metastatic melanoma. N Engl J Med. (2010) 363:809–19. 10.1056/NEJMoa1002011 20818844 PMC3724529

[49] Kopetz S Desai J Chan E Hecht JR O'Dwyer PJ Maru D. Phase II pilot study of vemurafenib in patients with metastatic BRAF-mutated colorectal cancer. J Clin Oncol. (2015) 33:4032–8. 10.1200/JCO.2015.63.2497 26460303 PMC4669589

[50] Prahallad A Sun C Huang S Di Nicolantonio F Salazar R Zecchin D. Unresponsiveness of colon cancer to BRAF(V600E) inhibition through feedback activation of EGFR. Nature. (2012) 483:100–3. 10.1038/nature10868 22281684

[51] Corcoran RB Ebi H Turke AB Coffee EM Nishino M Cogdill AP. EGFR-mediated re-activation of MAPK signaling contributes to insensitivity of BRAF mutant colorectal cancers to RAF inhibition with vemurafenib. Cancer Discov. (2012) 2:227–35. 10.1158/2159-8290.CD-11-0341 22448344 PMC3308191

[52] Tabernero J Grothey A Van Cutsem E Yaeger R Wasan H Yoshino T. Encorafenib plus cetuximab as a new standard of care for previously treated BRAF V600E-mutant metastatic colorectal cancer: updated survival results and subgroup analyses from the BEACON study. J Clin Oncol. (2021) 39:273–84. 10.1200/JCO.20.02088 33503393 PMC8078423

[53] Corcoran RB André T Atreya CE Schellens JHM Yoshino T Bendell JC. Combined BRAF, EGFR, and MEK inhibition in patients with BRAFV600E-mutant colorectal cancer. Cancer Discov. (2018) 8:428–43. 10.1158/2159-8290.CD-17-1226 29431699 PMC5882509

[54] Kopetz S Grothey A Yaeger R Van Cutsem E Desai J Yoshino T. Encorafenib, binimetinib, and cetuximab in BRAF V600E-Mutated colorectal cancer. N Engl J Med. (2019) 381:1632–43. 10.1056/NEJMoa1908075 31566309

[55] Sartore-Bianchi A Trusolino L Martino C Bencardino K Lonardi S Bergamo F. Dual-targeted therapy with trastuzumab and lapatinib in treatment-refractory, KRAS codon 12/13 wild-type, HER2-positive metastatic colorectal cancer (HERACLES): a proof-of-concept, multicentre, open-label, phase 2 trial. Lancet Oncol. (2016) 17:738–46. 10.1016/S1470-2045(16)00150-9 27108243

[56] Siena S Di Bartolomeo M Raghav K Masuishi T Loupakis F Kawakami H. Trastuzumab deruxtecan (DS-8201) in patients with HER2-expressing metastatic colorectal cancer (DESTINY-CRC01): a multicentre, open-label, phase 2 trial. Lancet Oncol. (2021) 22:779–89. 10.1016/S1470-2045(21)00086-3 33961795

[57] Takegawa N Yonesaka K. HER2 as an emerging oncotarget for colorectal cancer treatment after failure of anti-epidermal growth factor receptor therapy. Clin Colorectal Cancer. (2017) 16:247–51. 10.1016/j.clcc.2017.03.001 28363756

[58] Sartore-Bianchi A Amatu A Porcu L Ghezzi S Lonardi S Leone F. HER2 positivity predicts unresponsiveness to EGFR-targeted treatment in metastatic colorectal cancer. Oncologist. (2019) 24:1395–402. 10.1634/theoncologist.2018-0785 30952821 PMC6795149

[59] Feng Y Li Y Huang D Cai S Peng J. HER2 as a potential biomarker guiding adjuvant chemotherapy in stage II colorectal cancer. Eur J Surg Oncol. (2019) 45:167–73. 10.1016/j.ejso.2018.10.059 30420187

[60] Jung Y-S Park J-I. Wnt signaling in cancer: therapeutic targeting of Wnt signaling beyond β-catenin and the destruction complex. Exp Mol Med. (2020) 52:183–91. 10.1038/s12276-020-0380-6 32037398 PMC7062731

[61] Song L Li Y He B Gong Y. Development of small molecules targeting the wnt signaling pathway in cancer stem cells for the treatment of colorectal cancer. Clin Colorectal Cancer. (2015) 14:133–45. 10.1016/j.clcc.2015.02.001 25799881

[62] Aghabozorgi AS Bahreyni A Soleimani A Bahrami A Khazaei M Ferns GA. Role of adenomatous polyposis coli (APC) gene mutations in the pathogenesis of colorectal cancer; current status and perspectives. Biochimie. (2019) 157:64–71. 10.1016/j.biochi.2018.11.003 30414835

[63] Tanaka N Mashima T Mizutani A Sato A Aoyama A Gong B. APC mutations as a potential biomarker for sensitivity to tankyrase inhibitors in colorectal cancer. Mol Cancer Ther. (2017) 16:752–62. 10.1158/1535-7163.MCT-16-0578 28179481

[64] Takagawa R Fujii S Ohta M Nagano Y Kunisaki C Yamagishi S. Preoperative serum carcinoembryonic antigen level as a predictive factor of recurrence after curative resection of colorectal cancer. Ann Surg Oncol. (2008) 15:3433–9. 10.1245/s10434-008-0168-8 18846401

[65] Spindler BA Bergquist JR Thiels CA Habermann EB Kelley SR Larson DW. Incorporation of CEA improves risk stratification in stage II colon cancer. J Gastrointest Surg. (2017) 21:770–7. 10.1007/s11605-017-3391-4 28290141

[66] Lim KHT Kong HL Chang KTE Tan DSW Tan IBH Mohamad F. Recommended testing algorithms for NTRK gene fusions in pediatric and selected adult cancers: Consensus of a Singapore task force. Asia Pac J Clin Oncol. (2021) 18:394–403. 10.1111/ajco.13727 34806337 PMC9541932

[67] Pu XH Gao FP Wu HY Fu Y Fan XS. Correlation of NTRK genetic fusions with mismatch repair protein deletion in patients with colorectal cancer. Zhonghua Bing Li Xue Za Zhi. (2022) 51:103–7. 35152627 10.3760/cma.j.cn112151-20210716-00512

[68] Marcus L Donoghue M Aungst S Myers CE Helms WS Shen G. FDA approval summary: entrectinib for the treatment of NTRK gene fusion solid tumors. Clin Cancer Res. (2021) 27:928–32. 10.1158/1078-0432.CCR-20-2771 32967940

[69] Guinney J Dienstmann R Wang X de Reyniès A Schlicker A Soneson C. The consensus molecular subtypes of colorectal cancer. Nat Med. (2015) 21:1350–6. 26457759 10.1038/nm.3967 PMC4636487

[70] Becht E de Reyniès A Giraldo NA Pilati C Buttard B Lacroix L. Immune and stromal classification of colorectal cancer is associated with molecular subtypes and relevant for precision immunotherapy. Clin Cancer Res. (2016) 22:4057–66. 10.1158/1078-0432.CCR-15-2879 26994146

[71] Tauriello DVF Palomo-Ponce S Stork D Berenguer-Llergo A Badia-Ramentol J Iglesias M. TGFβ drives immune evasion in genetically reconstituted colon cancer metastasis. Nature. (2018) 554:538–43. 10.1038/nature25492 29443964

[72] Mooi JK Wirapati P Asher R Lee CK Savas P Price TJ. The prognostic impact of consensus molecular subtypes (CMS) and its predictive effects for bevacizumab benefit in metastatic colorectal cancer: molecular analysis of the AGITG MAX clinical trial. Ann Oncol. (2018) 29:2240–6. 10.1093/annonc/mdy410 30247524

[73] Li Y Yao Q Zhang L Mo S Cai S Huang D. Immunohistochemistry-based consensus molecular subtypes as a prognostic and predictive biomarker for adjuvant chemotherapy in patients with stage II colorectal cancer. Oncologist. (2020) 25:e1968–79. 10.1002/ONCO.13521 32926498 PMC8186407

[74] Allen WL Dunne PD McDade S Scanlon E Loughrey M Coleman H. Transcriptional subtyping and CD8 immunohistochemistry identifies poor prognosis stage II/III colorectal cancer patients who benefit from adjuvant chemotherapy. JCO Precis Oncol. (2018) 13:2018. 10.1200/PO.17.00241 30088816 PMC6040635

[75] Cohen SJ Punt CJA Iannotti N Saidman BH Sabbath KD Gabrail NY. Relationship of circulating tumor cells to tumor response, progression-free survival, and overall survival in patients with metastatic colorectal cancer. J Clin Oncol. (2008) 26:3213–21. 10.1200/JCO.2007.15.8923 18591556

[76] Krebs MG Metcalf RL Carter L Brady G Blackhall FH Dive C. Molecular analysis of circulating tumour cells-biology and biomarkers. Nat Rev Clin Oncol. (2014) 11:129–44. 10.1038/nrclinonc.2013.253 24445517

[77] Kidess E Heirich K Wiggin M Vysotskaia V Visser BC Marziali A. Mutation profiling of tumor DNA from plasma and tumor tissue of colorectal cancer patients with a novel, high-sensitivity multiplexed mutation detection platform. Oncotarget. (2015) 6:2549–61. 10.18632/oncotarget.3041 25575824 PMC4385870

[78] Siravegna G Mussolin B Buscarino M Corti G Cassingena A Crisafulli G. Clonal evolution and resistance to EGFR blockade in the blood of colorectal cancer patients. Nat Med. (2015) 21:795–801. 10.1038/nm.3870 26030179 PMC4868598

[79] Baxter NN Kennedy EB Bergsland E Berlin J George TJ Gill S. Adjuvant therapy for stage II colon cancer: ASCO guideline update. J Clin Oncol. (2022) 40:892–910. 10.1200/JCO.21.02538 34936379

[80] Tie J Wang Y Tomasetti C Li L Springer S Kinde I. Circulating tumor DNA analysis detects minimal residual disease and predicts recurrence in patients with stage II colon cancer. Sci Transl Med. (2016) 8:346ra92. 10.1126/scitranslmed.aaf6219 27384348 PMC5346159

[81] Tie J Kinde I Wang Y Wong HL Roebert J Christie M. Circulating tumor DNA as an early marker of therapeutic response in patients with metastatic colorectal cancer. Ann Oncol. (2015) 26:1715–22. 10.1093/annonc/mdv177 25851626 PMC4511218

[82] Parikh AR Van Seventer EE Siravegna G Hartwig AV Jaimovich A He Y. Minimal residual disease detection using a plasma-only circulating tumor DNA assay in patients with colorectal cancer. Clin Cancer Res. (2021) 27:5586–94. 10.1158/1078-0432.CCR-21-0410 33926918 PMC8530842

[83] Tie J Wang Y Cohen J Li L Hong W Christie M. Circulating tumor DNA dynamics and recurrence risk in patients undergoing curative intent resection of colorectal cancer liver metastases: A prospective cohort study. PLoS Med. (2021) 18:e1003620. 10.1371/journal.pmed.1003620 33939694 PMC8128260

[84] Reinert T Henriksen TV Christensen E Sharma S Salari R Sethi H. Analysis of plasma cell-free DNA by ultradeep sequencing in patients with stages I to III colorectal cancer. JAMA Oncol. (2019) 5:1124–31. 10.1001/jamaoncol.2019.0528 31070691 PMC6512280

[85] Bedin C Enzo MV Del Bianco P Pucciarelli S Nitti D Agostini M. Diagnostic and prognostic role of cell-free DNA testing for colorectal cancer patients. Int J Cancer. (2017) 140:1888–98. 10.1002/ijc.30565 27943272

